# *NGF* (*−198C > T, Ala35Val*) and *p75*^*NTR*^ (*Ser205Leu*) gene mutations are associated with liver function in different histopathological profiles of the patients with chronic viral hepatitis in the Brazilian Amazon

**DOI:** 10.1186/s10020-019-0134-x

**Published:** 2020-01-29

**Authors:** Leonn Mendes Soares Pereira, Ednelza da Silva Graça Amoras, Simone Regina Souza da Silva Conde, Sâmia Demachki, Eduardo José Melo dos Santos, Sandra Souza Lima, Ricardo Ishak, Antonio Carlos Rosário Vallinoto

**Affiliations:** 1 Virology Laboratory, Biological Science Institute, Federal University of Pará, Belém, Pará Brazil; 2 Postgraduate Program in Biology of Infectious and Parasitic Agents, Biological Science Institute, Federal University of Pará, Belém, Pará Brazil; 3 School of Medicine, Health Science Institute, Federal University of Pará, Belém, Pará Brazil; 4 Laboartory of Human and Medical Genetics, Biological Science Institute, Federal University of Pará, Belém, Pará Brazil

**Keywords:** Neurotrophins, Polymorphisms, Brazilian Amazon, Liver diseases, Hepatic physiopathology

## Abstract

**Backgrounds:**

Neural growth factor (NGF) is a neurotrophin that can interact with the p75^NTR^ receptor and initiate a cascade of reactions that determines cell survival or death, and both are associated with the physiology of liver tissue. Single nucleotide polymorphisms (SNPs) in the NGF and p75^NTR^ genes have been investigated in different pathologies; however, there are no studies that have analyzed their biological roles in the hepatic microenvironment. In the present study, we evaluated the impact of SNPs in these genes on the maintenance of liver function at different stages of inflammation and fibrosis in patients with chronic viral liver disease in the Brazilian Amazon.

**Methods:**

The SNPs *-198C > T, Arg80Gln*, *Val72Met*, *Ala35Val*, *Ala18Ala* and *Ser205Leu* were genotyped by real-time PCR in samples from patients with chronic viral hepatitis stratified by stage of inflammation and liver fibrosis. Histopathological, viral load (VL), liver enzyme and comorbidities data were obtained from updated medical records. Other aspects were highlighted by applied epidemiological questionnaires.

**Results:**

The *-198C/T* and *Ala35Val* polymorphisms in NGF were associated with changes in histopathological profiles, VL and liver enzymes. *Ser205Leu* polymorphism in p75^NTR^ was associated only with changes in VL and liver enzymes. Polymorphic frequencies were variable among different ethnic populations, mainly for biologically relevant polymorphisms. A multifactorial network of interactions has been established based on genetic, virological, behavioral and biochemical aspects.

**Conclusion:**

Mutations in the NGF *(−198C > T*, *Ala35Val)* and p75^NTR^ (*Ser205Leu*) genes, within the list of multifactorial aspects, are associated with liver function in different histopathological profiles of patients with chronic viral liver disease in the Brazilian Amazon.

## Introduction

Neural growth factor (NGF) is a neurotrophin with two non-covalently bound monomers. Each monomer has four loop regions with polar amino acid sequences and two β-pleated sheet strands (Mcdonald et al., 1991). Classically, the mature form of the peptide is associated with neural stimulation and survival (Cohen-Cory et al., 1991).

A range of receptors may interact with NGF. Among them, the p75^NTR^ receptor is a low-affinity transmembrane protein that contains a cysteine-rich extracellular domain and an intracellular domain with a palmitoylation site and serine and threonine residues (Baldwin et al., 1992). The receptor can promote cell survival when it interacts with receptors of the tyrosine kinase (Trk) family, leading to activation of the phosphoinositide 3-kinase - protein kinase β (PI3K-AKT) cascade or activation of the Fas apoptosis inhibitory molecule (FAIM) and MAP kinase (MAPK) pathways (Eibl et al., 2012; Roux and Barker, 2002). In the absence of Trk, p75^NTR^ is proapoptotic and activates the c-Jun N-terminal kinase (JNK) pathway (Bhakar et al., 2003; Nykjaer et al., 2005). However, this pathway requires coexpression of the factors neurotrophin receptor interacting factor (NRIF) and tumor necrosis factor receptor (TNFR)-associated factor 6 (TRAF6), which can also favor cell survival through nuclear factor kappa β (NF-kB) activation (Gentry, et al., 2004).

With the improvement in molecular detection techniques, the expression of NGF and p75^NTR^ in non-neuronal cell lines could be observed. In the hepatic microenvironment, NGF is expressed in damaged hepatocytes, exerts an apoptotic effect on fibrogenic cells via p75^NTR^ (Oakley et al., 2003) and upregulates hepatoprotective components in cholestatic liver tissue (Tsai et al., 2018), suggesting that neurotrophins participate in the control of liver injury, as observed in cirrhotic tissues (Cassiman et al., 2001); however, other conclusions have been inferred when analyzing different neurotrophin receptors (Rasi et al., 2007), ligands (Kendall et al., 2009) and histological stages of the liver (Amoras et al., 2015).

Despite the knowledge available on the dynamics of neurotrophins in liver tissue, there are no studies that have evaluated genetic variations in these factors as drivers. However, the biological significance of single nucleotide polymorphisms (SNPs) in NGF and p75^NTR^ has been investigated in other pathologies.

The *-198C > T* polymorphism (rs11102930) located in the promoter region of the *NGF* gene has been associated with multiple sclerosis (Akkad et al., 2008), childhood IgA neuropathy (Hahn et al., 2011) and asthmatic disease (Szczepankiewicz et al., 2012). These studies propose that substitution of cytosine with thymine in that specific position (nt − 198) modifies the binding site for transcription factors such as vitamin D receptor (VDR) and specificity protein 1 (Sp1), which in turn alters NGF expression.

SNPs located in the 3rd exon are relevant due to their importance in the encoding of NGF. The polymorphism *+ 273C > T* (rs6330) is characterized by the substitution of a cytosine with a thymine at position + 273 of the exon, which causes the change in the amino acid alanine to valine (*Ala35Val*) at position 35 of the peptide (Cozza et al., 2008). The increase in the molar mass generated by the change in amino acids modifies the tertiary structure of the protein and leads to changes in signaling, which in multiple sclerosis represents a protective factor (Hahn et al., 2011). The T allele was also considered neuroprotective in patients with Alzheimer’s disease (Nagata et al., 2011) and a predictor of efficacy of cognitive behavioral therapy in children with anxiety (Lester et al., 2012).

In regard to the polymorphisms *Ala18Ala* (rs6325), *Val72Met* (rs11466110) and *Arg80Gln* (rs11466111), also located in exon 3, it is still unclear what changes they cause because the frequency of the most rare alleles (MAF) is less than 5%, which hinders genotypic analyses (Di Maria et al., 2012; Levran et al., 2012).

For the p75^NTR^ receptor, the *Ser205Leu* polymorphism (rs2072446) consists of the substitution of cytosine with thymine in exon 6, resulting in the amino acid serine being replaced by leucine in codon 205, a conserved intracellular region rich in serine and threonine residues where O-linked glycosylation occurs (Taniuchi et al., 1986; Chapman et al., 1996). The change in the peptide is related to structural changes, cellular localization and receptor signaling (Cohen-Cory et al., 1991; Drysdale et al., 2000). This SNP is associated with depressive disorder in Japanese women (Fujii et al., 2011) and Alzheimer’s disease (Lester et al., 2012), and the *Leu* variant has a protective role against the development of these disorders, although heterozygous patients have a weaker response to antidepressants than do patients homozygous for *Ser*^*+/+*^ (Gau et al., 2008). The selection of SNPs representative of haplotypes (*tag* SNPs) is being used as a way of representing the biological significance of the genetic variations of p75^NTR^ (Wang et al., 2014).

Due to the lack of studies on the role of SNPs in NGF and p75^NTR^ in liver tissue, the present study is the first to identify genetic variations as factors that impact liver function at different stages of inflammation and tissue fibrosis in a miscegenated population. These findings are expected to contribute to the knowledge regarding the functions and mechanisms of action of neurotrophins and their receptors in different microenvironments.

## Materials and methods

### Study population and ethical aspects

This is a cross-sectional and analytical study developed in partnership with the Fundação Santa Casa de Misericórdia do Pará (Santa Casa de Misericórdia Foundation of the State of Pará - FSCMPA), João de Barros Barreto University Hospital (HUJBB) and the Virology Laboratory of the Biological Science Institute of the Federal University of Pará (LABVIR-ICB- UFPA) between 2014 and 2017.

Screening for inclusion was based on clinical and laboratory results as previously described (Pereira et al., 2018): Patients with persistent HBsAg for more than 6 months, positive or negative HBeAg, positive anti-HBeAg, and clinical and histological changes were included among patients with chronic hepatitis B (PCHB). Patients characterized by clinical and histological changes, variations in serum liver enzyme levels and positive viral load for *Hepacivirus C* were included in the group of patients with chronic hepatitis C (PCHC). Both groups of infected were not on specific therapy. Were obtained 35 PCHB and 68 PCHC in this study.

A control group (CG) of 300 blood donors from the Center for Hemotherapy and Hematology of Pará Foundation (HEMOPA), seronegative, and undetectable viral load for HBV, *Hepacivirus C*, and other agents typically screened in blood bank screening were determined. This group is specifically used to compare the genetic frequency of the studied polymorphisms (Pereira et al., 2018).

The frequency of polymorphic variants in different ethnic populations was obtained from databases containing public access catalogs of human genotypes available in the National Center for Biotechnology Information (NCBI). Data from a heterogeneous global population (Global), South Americans (SA), African Americans (AA), Native Americans (NA), Europeans (EUR), Africans (AFR) and Asians (ASI) were included (https://www.ncbi.nlm.nih.gov/snp/).

The present study was submitted to and approved by the Research Ethics Committee of the FSCMPA, under protocols no. 117/2009 and 684,432/2014, following the Human Research Guidelines and Standards (Resolution 196 of the Brazilian National Health Council). All individuals who agreed to participate in the study signed an informed consent form. Subsequently, they answered the project’s epidemiological questionnaire in order to obtain their demographic, social and behavioral information.

### Clinical, biochemical, virological and histological data collection

The presence of comorbidities, the levels of liver enzymes (alanine aminotransferase [ALT], aspartate aminotransferase [AST], and gamma-glutamyl transferase [GGT]) and plasma viral load (VL) of HBV and *Hepacivirus C* were obtained from updated medical records.

Liver biopsies were performed when recommended by a qualified medical board, following a specific clinical protocol under the jurisdiction of the Brazilian public health system, which treats this information with extreme confidentiality. After authorization was provided, the data of interest were collected from the medical records of and medical interviews with individuals who agreed to participate in the study.

Histopathological profiles were established at the Pathology Anatomy Department of the Federal University of Pará, based on the METAVIR classification. Stage A0-A1 was assigned when inflammation was absent or mild, and stage A2-A3 was assigned when inflammation was moderate or severe. Fibrosis was classified as F0-F1 in the absence of liver parenchymal abnormalities or presence of portal fibrosis without septa, as F2 in the presence of portal fibrosis with rare septa and as F3-F4 when there were numerous septa or liver cirrhosis.

### Molecular analyses of polymorphisms

A 5 mL sample of peripheral blood was collected in vacutainer EDTA tubes. The samples were centrifuged at 5000 rpm to separate the plasma, leukocyte and erythrocyte fractions. Genomic DNA was extracted from the leukocytes following a protocol previously described by Cigliero et al., 2011.

For genotyping the *NGF* and *p75*^*NTR*^ gene polymorphisms, real-time PCR (qPCR) with a StepOne PLUS Sequence Detector (AppliedBiosystems, Foster City, CA, USA) was used. TaqMan® SNP Genotyping Assays and the following primer sequences were used: ***NGF***: ***-198C > T*** -C_26680904_10; ***Arg80Gln*** -C_25619679_10; ***Val72Met*** -C_25619678_10; ***Ala35Val*** -C_2525309_10; ***Ala18Ala*** -C_12072709_10; and ***p75***^***NTR***^: ***Ser205Leu*** -C_15870920_10 (Applied Biosystems, Foster City, CA, USA). Each reaction contained 7 μl of distilled water, 10 μl of Universal PCR Master Mix (2X) (Applied Biosystems, Foster City, CA, USA), 1 μL of TaqMan® Assay Buffer (20X) (Applied Biosystems, Foster City, CA, USA) and 2 μL of extracted DNA. The following conditions were used for amplification: 60 °C for 30 s; 95 °C for 10 min; 50 cycles of 92 °C for 30 s and 60 °C for 1 min and 30 s.

### Statistical analyses

Bivariate analyses were performed to investigate the factors associated with liver inflammation and fibrosis. Sex was evaluated using Fisher’s exact test and the chi-square test. VL was analyzed using the Mann-Whitney and Kruskal-Wallis tests. Liver enzyme levels were categorized based on reference values provided with the quantification kits used (34 UI/L for ALT; 55 UI/L for AST; 64 UI/L in males and 36 UI/L in females for GGT) (Clinical Chemistry- ARCHITEC/AEROSET, ABBOTT) and were subsequently analyzed according to Fisher’s exact test and the G test. The alcoholism and presence of comorbidities were also assessed by G-test and Fisher’s exact test.

**Fig. 1:**
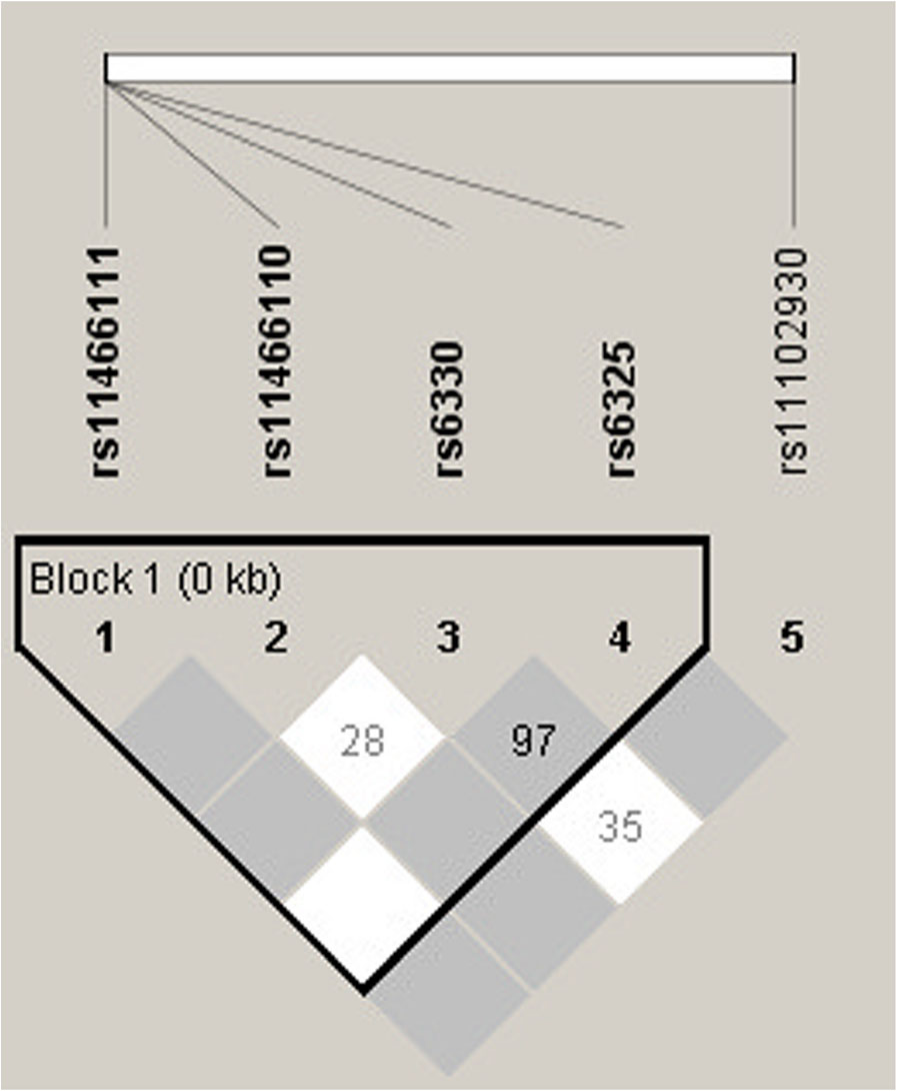
Linkage disequilibrium between *NGF* gene polymorphisms. The horizontal white bar symbolizes the location of the polymorphisms in the gene. Only normalized linkage disequilibrium values below 1 are shown (10x scale). One haplotype block was inferred among the polymorphisms located in exon 3 of the gene

Based on the confidence interval of the normalized linkage disequilibrium coefficient (D’), a haplotype block was inferred for polymorphisms in exon 3 of NGF, using the software Haploview 4.2 (Fig. 1). Hardy-Weinberg equilibrium was assessed using the chi-square test. The frequency of polymorphic variants was determined by direct count and compared among the histological profiles by the G and chi-square tests. Chi-square residue analysis was applied to determine which frequencies varied from expected.

For significant data, the dependence between the biological factors and the liver histological profile was calculated through simple and ordinal logistic regression.

The liver enzyme levels and VL were correlated through Pearson’s linear matrix and compared among the polymorphic variants by the Mann-Whitney test. Heatmap grouping plots were proposed based on normalized data of VL and liver enzyme, according to the histopathological profile and the polymorphic variants investigated.

Statistical calculations were performed using BioEstat 5.0 (Ayres et al., 2008), GraphPad Prism version 6.1 and Minitab 14 software, adopting a significance level of 5% (*p* ≤ 0.05).

An interaction network between the clinical, virological, biochemical, histological and genetic factors was constructed with the statistical data generated using Cytoscape 3.6 software and based on the recommendations of Taylor, 1990.

## Results

### Clinical, biochemical and virological factors associated with the risk of hepatic microenvironment changes

Male individuals (60.19%), the PCHC group (66.02%) and milder histological profile stages predominated in all groups analyzed. A relatively close proportion was observed between the F0-F1 and F2-F3 classifications in the PCHC group (39.71 and 35.29%, respectively). There was a proportional distribution between alcoholics and those who did not have this behavior. Comorbidities were frequent in the advanced histological profiles. The average value of liver enzymes increased with scores of inflammation and hepatic fibrosis (Table 1).

**Table 1:** Characterization of stratified groups according to METAVIR classification

METAVIR	Genre		Populations (%)		Ethylism		Comorbidities		V.L. (*log*^*10*^)^a^	ALT^a^	AST*	GGT^a^
	M	F	PHCC	PHBC	No	Yes	No	Yes				
Degree of hepatic inflammation												
A0-A1	43	26	40 (58.82)	29 (82.86)	36 (72.00)	33 (66.00)	33 (78.57)	36 (59.02)	5.63 ± 0.74	62.52 ± 60.48	62.52 ± 60.48	63.19 ± 74.28
A2-A3	19	15	28 (41.18)	06 (17.14)	14 (28.00)	17 (34.00)	9 (21.43)	25 (40.98)	5.21 ± 1.15	106.93 ± 74.84	90.23 ± 71.84	89.33 ± 60.93
Degree of liver fibrosis												
F0-F1	29	20	27 (39.71)	22 (62.86)	26 (52.00)	23 (46.00)	29 (69.05)	20 (32.79)	5.31 ± 1.00	72.53 ± 71.50	55.62 ± 40.16	66.68 ± 74.94
F2	16	10	17 (25.00)	09 (25.71)	13 (26.00)	14 (28.00)	4 (09.52)	23 (37.70)	5.63 ± 0.64	74.88 ± 63.10	62.77 ± 47.64	62.79 ± 47.43
F3-F4	17	11	24 (35.29)	04 (11.43)	11 (22.00)	13 (26.00)	9 (21.43)	18 (29.51)	5.43 ± 1.14	87.96 ± 69.11	82.87 ± 74.6	91.90 ± 80.45
Total	62	41	68	35	50	50	42	61	5.45 ± 0.96	76.86 ± 68.33	64.00 ± 52.69	72.19 ± 70.75

^a^ - Mean ± standard deviation values; *PHCC* Patients with chronic hepatitis C, *PHBC* Patients with chronic hepatitis B, *V.L.* Viral Load

Regarding liver inflammation, individuals with elevated ALT levels were primarily classified as inflammatory stage A2-A3, with a risk of ALT levels increasing 20-fold in patients with high inflammatory activity. Similarly, the increase in AST and GGT levels was higher in patients classified with moderate/severe inflammatory activity, with an approximately 5 and 7-fold risk, respectively, of changes in the levels of these markers being related to liver inflammation (Tables 2 and 3).

**Table 2:** Bivariate analysis of risk factors related to inflammation and hepatic fibrosis classified according to the METAVIR scale

FACTORS	Degree of hepatic inflammation					Degree of liver fibrosis					
	A0-A1	A2-A3	*P*	Odds ratio	CI 95%	F0-F1	F2	F3-F4	*P*	Odds ratio	CI 95%
GENDER	n (%)	n (%)				n (%)	n (%)	n (%)			
Male	43 (62.32)	19 (55.88)	0.67^♠^			29 (59.18)	16 (61.54)	17 (60.71)	0.98^**Ω**^		
Female	26 (37.68)	15 (44.12)				20 (40.82)	10 (38.46)	11 (39.29)			
VL (log_10_) (median)	5.4109	5.3664	0.65^♦^			4.4402	5.6433	5.6503	< 0.05^**Ψ**^	1.97^*^	0.34–0.75
ALT											
≤ 34 UI/L	26 (40.00)	1 (03.23)	< 0.01^♠^	20.00^#^	1.57–155.87	20 (42.55)	4 (15.38)	3 (13.04)	0.01^■^	9.74^**#**^	1.16–81.69
> 34 UI/L	39 (60.00)	30 (96.77)				27 (57.45)	22 (84.66)	20 (86.96)			
AST											
≤ 55 UI/L	44 (67.69)	10 (31.25)	< 0.01^♠^	4.61^#^	1.85–11.46	30 (62.50)	15 (57.69)	9 (30.13)	0.17^■^		
> 55 UI/L	21 (32.31)	22 (68.75)				18 (37.50)	11 (42.31)	14 (60.87)			
GGT											
≤ 64 UI/L (♂) ≤36 UI/L (♀)	41 (67.21)	7 (21.88)	< 0.01^♠^	7.32^#^	2.71–19.78	26 (57.78)	12 (50.00)	10 (41.67)	0.44^■^		
> 64 UI/L (♂) > 36 UI/L (♀)	20 (32.79)	25 (78.12)				19 (42.22)	12 (50.00)	14 (58.33)			
Ethylism											
No	36 (72.00)	14 (28.00)	0.65^♠^			26 (52.00)	13 (26.00)	11 (22.00)	0.83^■^		
Yes	33 (66.00)	17 (34.00)				23 (46.00)	14 (28.00)	13 (26.00)			
Comorbidities											
No	33 (78.57)	9 (21.43)	0.04^♠^	2.55^#^	1.04–6.24	29 (69.05)	4 (09.52)	9 (21.43)	< 0.01^■^	1.63^*^	–
Yes	36 (59.02)	25 (40.98)				20 (32.79)	23 (37.70)	18 (29.51)

**Table 3:** Bivariate analysis of risk factors related to inflammation and hepatic fibrosis classified according to the METAVIR scale

Evaluation of polymorphisms	A0-A1	A2-A3	CG	*P*	Odds ratio	CI 95%	F0-F1	F2	F3-F4	CG	*P*	Odds ratio	CI 95%
*NGF*													
*-198C > T*													
*CC*	14 (20.59)	3 (08.82)	94 (31.33)	0.01^d^	1.0^g^	–	11 (22.45)	2 (07.69)	4 (14.81)	94 (31.33)	0.04^d^	9.0^g^	–
*CT*	41 (60.29)	19 (55.88)	152 (50.67)				26 (53.06)	19 (73.08)	15 (55.56)	152 (50.67)			
*TT*	13 (19.12)	12 (35.29)	54 (18.00)				12 (24.49)	5 (19.23)	8 (29.63)	54 (18.00)			
*C*	69 (50.74)	25 (36.76)	340 (56.67)	0.00^a^			48 (48.98)	23 (44.23)	23 (42.59)	340 (56.67)	0.06 ^a^		
*T*	67 (49.26)	43 (63.24)	260 (43.33)				50 (51.02)	29 (55.77)	31 (57.41)	260 (43.33)			
HAPLOTYPES													
*ArgValAlaAla*^*+/+*^	29 (44.62)	11 (35.48)	100 (33.33)	0.00^d^	1.4^g^	–	17 (37.78)	11 (44.00)	12 (46.15)	100 (33.33)	0.00^**c**^	1.1^g^	–
*ArgValAlaAla* *ArgValValAla*	28 (43.08)	14 (45.16)	110 (36.73)				22 (48.89)	12 (48.00)	8 (30.77)	110 (36.73)			
*ArgValValAla*^*+/+*^	4 (06.15)	5 (16.13)	14 (04.76)				3 (06.67)	1 (04.00)	5 (19.23)	14 (04.76)			
Other haplotypes	4 (06.15)	1 (03,23)	76 (25.17)				3 (06.67)	1 (04.00)	1 (03.85)	76 (25.17)			
*Ser205Leu*													
*SerSer*	59 (89.39)	30 (90.91)	264 (88.00)	0.85^d^			45 (93.75)	25 (96.15)	19 (76.00)	264 (88.00)	0.70^d^		
*SerLeu*	6 (09.09)	3 (10.61)	35 (11.67)				3 (06.25)	5 (16.13)	1 (24.00)	35 (11.67)			
*LeuLeu*	1 (01.52)	0	1 (00.33)				0	1 (03.23)	0	1 (00.33)			
*Ser*	124 (93.94)	63 (95.45)	563 (93.83)	0.87^a^			93 (96.88)	55 (88.71)	39 (97.50)	563 (93.83)	0.17^d^		
*Leu*	8 (06.06)	3 (04.55)	37 (06.17)				3 (03.12)	7 (11.29)	1 (02.50)	37 (06.17)			

^a^ Fisher’s exact test; ^b^ Kruskal-Wallis tests; ^**c**^ Chi-square test; ^d^ G test; ^e^ Mann-Whitney test; ^f^ Simple logistic regression; ^g^ Ordinal logistic regression

For fibrosis, the VL was statistically lower in the early stages of parenchymal abnormalities, and an VL increase of 1 log_10_ increased the risk approximately 2-fold for patients to manifest more severe structural changes in liver tissue (Tables 2 and 3).

ALT levels were also altered in fibrosis, with a prevalence of patients with moderate enzyme levels classified as F0-F1, with an approximately 10-fold risk for elevated ALT to be associated with moderate fibrosis and cirrhosis (Tables 2 and 3).

These data indicate that ALT may be a biochemical factor that is sensitive to liver histological changes; however, the confidence intervals were wide, with values ranging between 1.57 to 155.87 for inflammation and 1.16 to 81.69 for fibrosis (Tables 2 and 3).

The alcoholism was not associated with histopathological profiles of liver tissue. Statistical relevance was observed for the presence of associated comorbidities; the chances of acquiring comorbidities increase by approximately 3 folds as the tissue progresses to elevated inflammation. In fibrosis, chances also increase as histological progression to more aggravating profiles occurs (Tables 2 and 3). In the present study, the most frequent comorbidity was Systemic Arterial Hypertension (SAH) (21.05%), followed by Diabetes Mellitus (DM) (7.02%). Complex multi-symptomatic clinical profiles with 3 or more comorbidities were frequent (14.04%), in which, besides SAH and DM, cases of chronic renal failure (CRF), hepatic steatosis, dyslipidemia, among others were observed (data not shown).

### Frequency of polymorphisms in NGF and p75^NTR^ according to histological stratifications

All polymorphisms studied in NGF and p75^NTR^ were in Hardy-Weinberg equilibrium. For the NGF polymorphism ***-****198C > T*, the frequency of the *CC* genotype was higher in patients classified as F0-F1 and F2. The heterozygous genotype *CT* exhibited the highest frequency among all histological stages of inflammation and fibrosis. The frequency of polymorphism was statistically different among infected patients compared to CG group, in which the frequency of *C* allele was higher than expected. The ordinal analysis showed that the probability of the individual carry the *C* allele is greater if it is healthier than in patients infected, especially when it comes to liver fibrosis (Tables 2 and 3).

Regarding the haplotypes in exon 3 of NGF, the most frequent blocks were homozygous *ArgValAlaAla*^*+/+*^, heterozygous *ArgValAlaAla - ArgValValAla* and homozygous *ArgValValAla*^*+/+*^. Among these, the block *ArgValValAla*^*+/+*^ was less homogeneous among all inflammation and fibrosis classifications. “Other haplotypes”, including the blocks *ArgValAlaAla, ArgMetAlaAla, ArgValAlaAla, ArgValValAla, ArgMetValAla, ArgMetValAla, ArgValAlaAla,* and *ArgValAlaAla*, were the least frequent in all groups analyzed, except for F0-F1 and F2 fibrosis, whose proportions were similar to those for the homozygous *ArgValValAla* block. It is suggested that the significant frequency of “other haplotypes” in the CG group is due to differences in prevalence of the variant *Arg80Gln* and *Val72Met* compared to infected patients (Tables 2, 4). Residual analysis showed that the frequency of variant *ArgValValAla*^*+/+*^ was higher than expected in patients with high inflammation and advanced fibrosis. In ordinal regression, considering variant *ArgValValAla*^*+/+*^ as a predictor of inflammation and fibrosis, the haplotype frequency was higher in patients with advanced histological changes.

**Table 4:** Frequency of polymorphisms in *NGF* and *p75*^*NTR*^ genes in different ethnic populations

			ETHNIC POPULATIONS									
Gene	SNP	Alleles	AB	AB	Global	SA	AA	NA	EUR	AFR	ASI	*P* < 0.05
*NGF*			**Ref.:**		*1000Genomes*	*The PAGE Study*	*The PAGE Study*	*The PAGE Study*	*1000Genomes*	*1000Genomes*	*The PAGE Study*	
	*-198C > T*	C	0.461	0.567	0.532	0.535	0.638	0.489	0.344	0.685	0.540	Yes
		T	0.539	0.433	0.468	0.465	0.362	0.511	0.656	0.315	0.460	
			**Ref.:**		*–*	*–*	*–*	*–*	*–*	*–*	*–*	
	*Arg80Gln*	*Arg*	0.995	0.889	0.995	0.994	0.996	0.986	0.988	1.00	1.00	Yes
		*Gln*	0.005	0.111	0.005	0.006	0.004	0.014	0.012	0.00	0.00	
			**Ref.:**		*–*	*–*	*–*	*–*	*–*	*–*	*–*	
	*Val72Met*	*Val*	0.981	1.00	0.993	0.999	0.979	0.995	0.999	0.975	1.00	Yes
		*Met*	0.019	0.00	0.007	0.001	0.121	0.005	0.001	0.025	0.00	
			**Ref.:**		*–*	*–*	*–*	*–*	*–*	*–*	*–*	
	*Ala35Val*	*Ala*	0.672	0.669	0.753	0.670	0.798	0.624	0.544	0.845	0.803	Yes
		*Val*	0.328	0.331	0.247	0.330	0.202	0.376	0.456	0.155	0.197	
			**Ref.:**		–	–	*GO Exome Sequencing Project*	–	–	–	*ExAc*	
	*Ala18Ala*	*Ala-*C	0.990	0.98	0.955	–	0.881	–	0.999	0.837	0.999	No
		*Ala-*T	0.010	0.02	0.045	–	0.119	–	0.001	0.163	0.001	
			**Ref.:**		–	–	*The PAGE Study*	–	–	–	*The PAGE Study*	
*p75*^*NTR*^	*Ser205Leu*	*Ser*	0.944	0.938	0.947	0.962	0.985	0.963	0.936	0.995	0.898	Yes
		*Leu*	0.056	0.062	0.053	0.038	0.015	0.037	0.064	0.005	0.102	

Marked cells have allele frequencies statistically similar to the infected group

*AB* Amazonian-Brazilian population infected. *AB* No infected, *SA* South American descendent population, *AA* African American descendent population, *NA* Native American descent population, *EUR* European descendent population, *AFR* African population, *ASI* Asian descendant population

The *SerSer* variant of the *Ser205Leu* polymorphism of p75^NTR^ was predominant in all groups analyzed. The *LeuLeu* variant was represented only in classifications A0-A1, F2 and CG. Significant differences were not observed in the comparison between the different groups analyzed (Tables 2 and 3).

The frequencies of the polymorphisms obtained in the present study were compared with a public access database available for different ethnicities evaluated. The group of infected patients and CG were treated as ancestors of the Brazilian Amazon population (“AB” **-** infected and “AB” - no infected, respectively). In the statistic, the frequency of *Ala18Ala* and *Arg80Gln* (in that order) were similar between different populations; for the *-198C > T* polymorphism, only the NA population was similar to the infected patients; and for the *Ala35Val* polymorphism similarities were observed between the uninfected group and the SA population (Table 4).

**Fig. 2:**
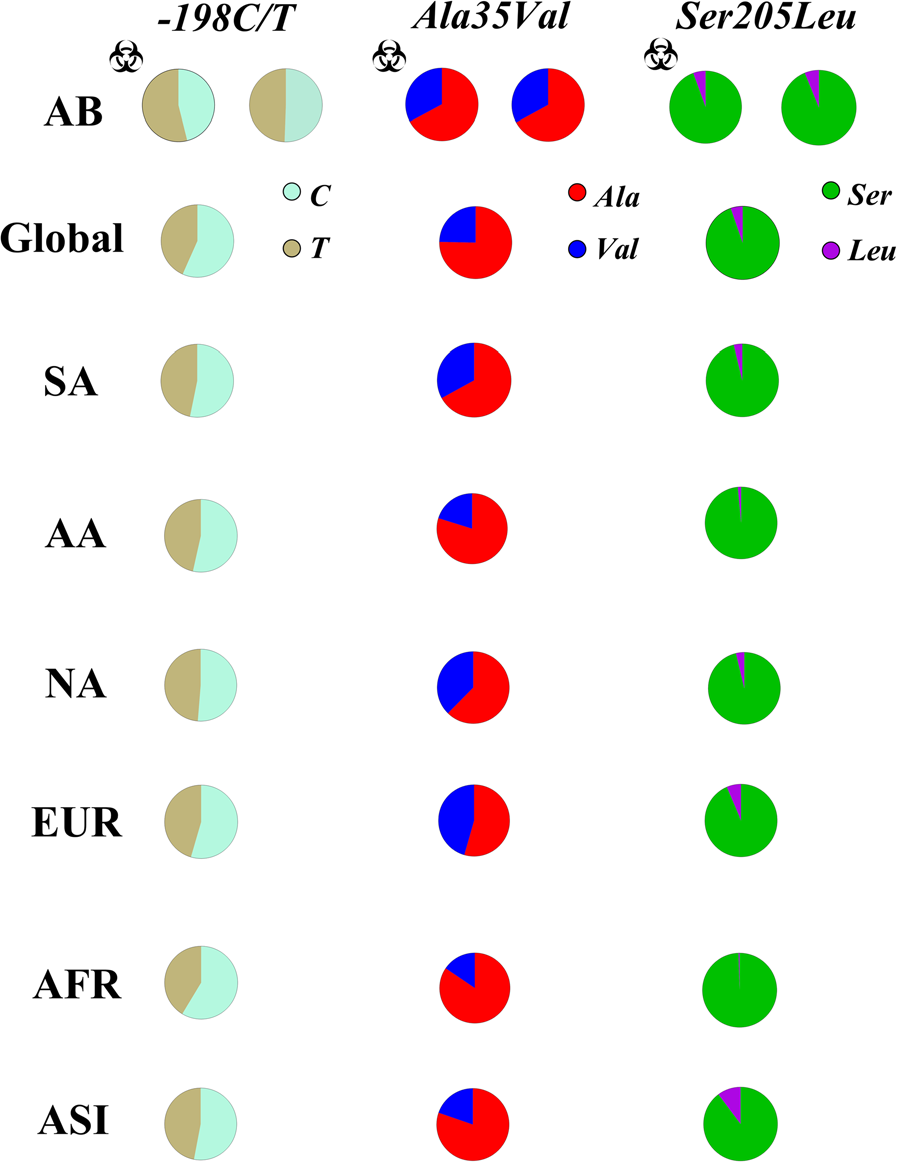
Frequency of biologically relevant polymorphisms (*−198C/T; Ala35Val; Ser205Leu*). The most frequent variants prevailed in the African population. MAF ranged between European (*−198C/T*) and Asian (*Ser205Leu*)

The universal frequency of clinically significance polymorphisms (presented later) were represented in “part of a whole” graphs (Fig. 2). The most frequent alleles were prevalent in the AFR population (*p > 0.01*), only for the *-198C/T* polymorphism the frequency was similar between AA and AB as well; MAF prevalence varied among ethnic populations (*p > 0.01*), for polymorphisms *-198C > T* and *Ala35Val* the predominance was in the EUR population, for polymorphism *Ser205Leu* this tendency was observed in ASI.

### Association of NGF and p75^NTR^ polymorphisms with liver enzyme levels and plasma VL

**Fig. 3:**
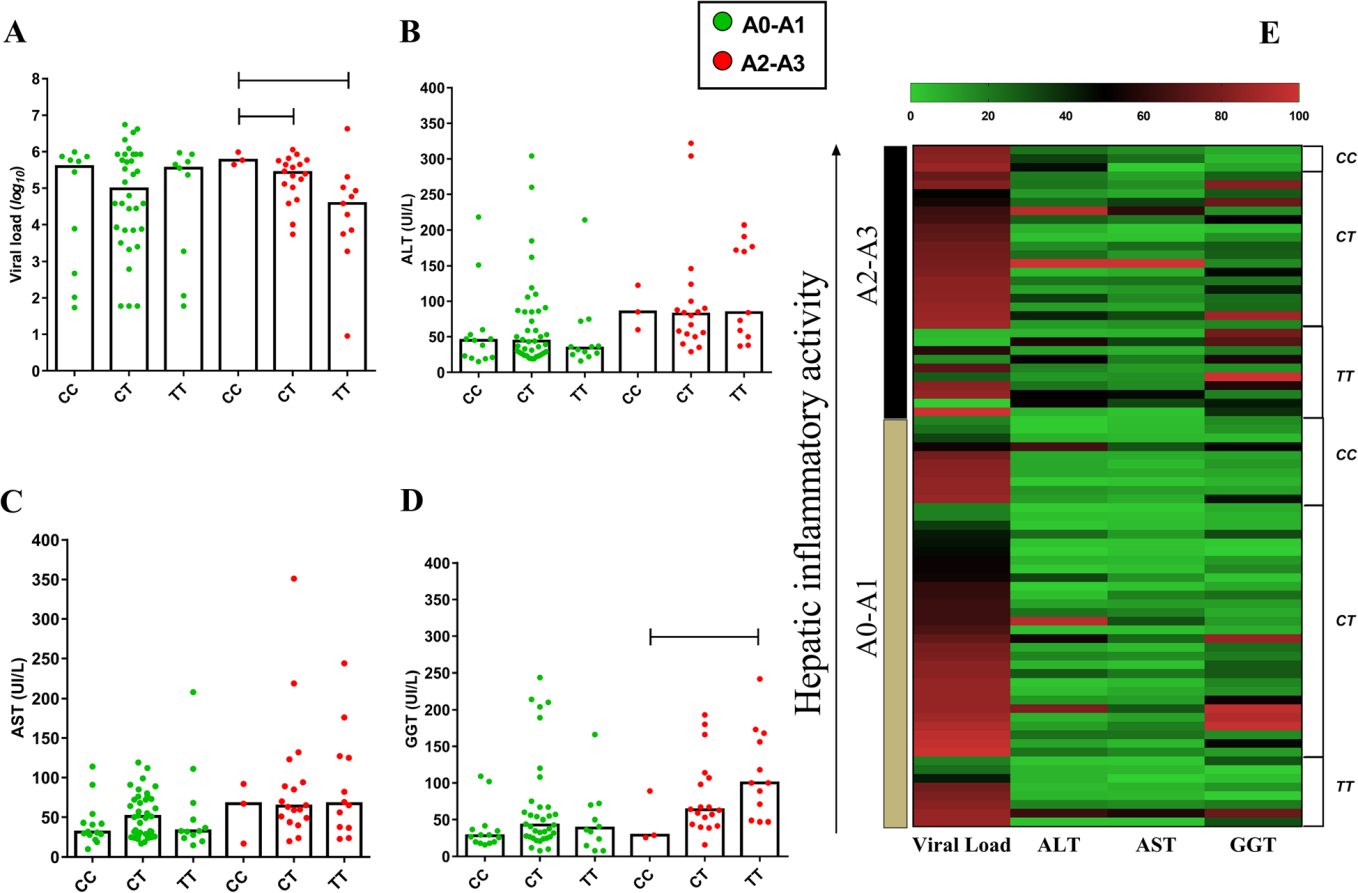
Association of liver function with -198C/T polymorphism according to hepatic inflammation. In the A2-A3 inflammatory profile, patients with the *CC* genotype had high plasma VL levels (**a**), ALT (**b**), AST (**c**) and low GGT levels (**d**). In the heatmap (**e**), high VL levels prevailed in all inflammation scores, however, for the *TT* genotype there is a tendency to group low VL levels in intense inflammation; in this same profile, the highest levels of liver enzymes are grouped

In the A2-A3 inflammatory profile, patients with the *CC* genotype (*−198C/T*) had high plasma VL levels (*CC* vs *CT - p: 0.0385; CC* vs *TT - p: 0.0108*) (Fig. 3a) and low GGT levels (*CC* vs *TT - p: 0.05*) (Fig. 3d). In the heatmap, high VL levels prevailed in all inflammation scores, however, for the *TT* genotype there is a tendency to group low VL levels in intense inflammation; in this same profile, the highest levels of liver enzymes are grouped (Fig. 3e).

**Fig. 4:**
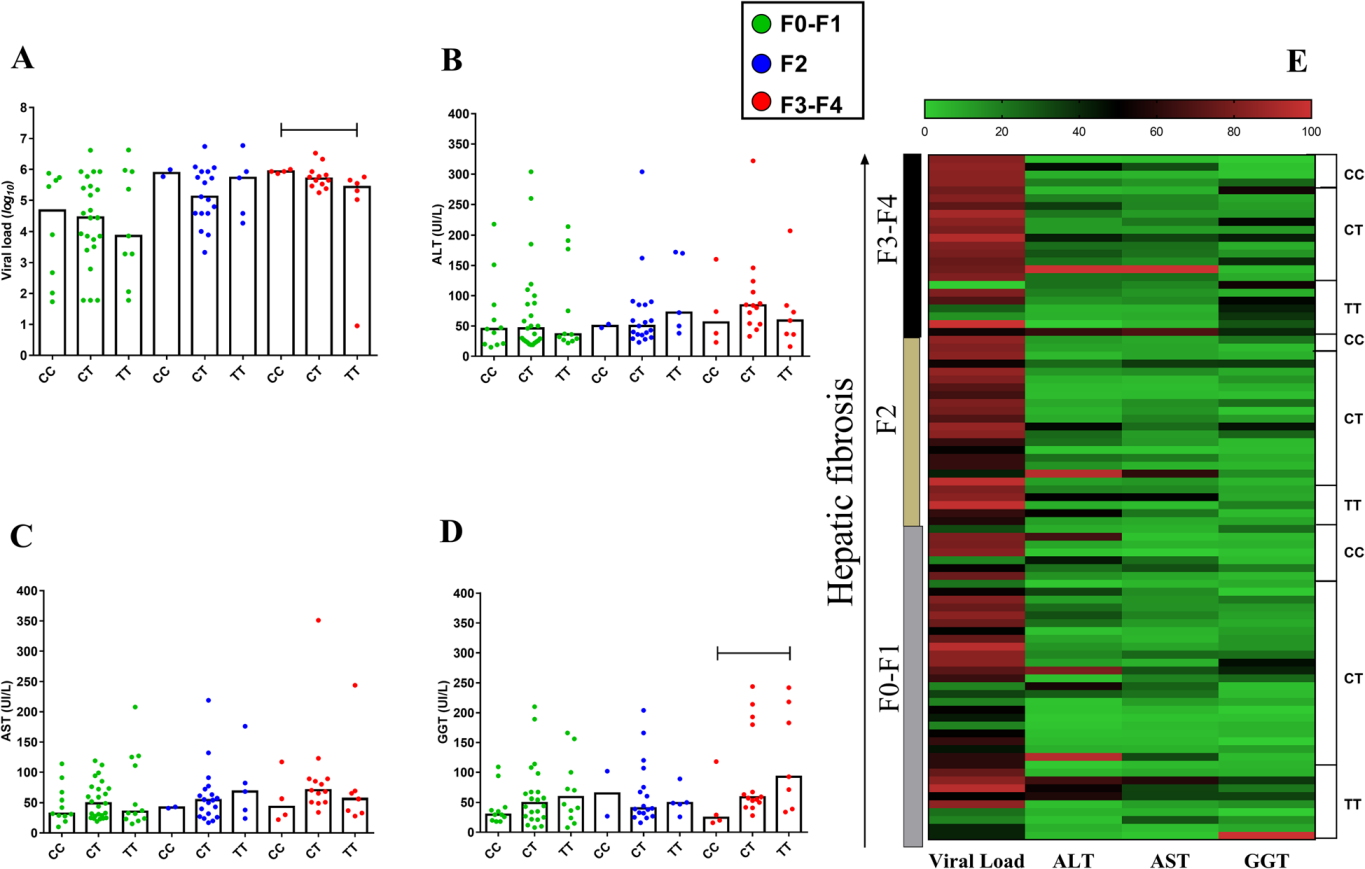
Association of liver function with -198C/T polymorphism according to hepatic fibrosis. In the F3-F4 fibrosis profile, patients with the *CC* genotype had high plasma VL levels (**a**), ALT (**b**), AST (**c**) and GGT levels (**d**). In the heatmap graph (**e**), no significant groupings were observed between the analyzed factors

Similar findings were obtained for *CC* genotype in patients with F3-F4 fibrosis for both VL (*CC* vs *TT - p: 0.014*) and GGT (*CC* vs *TT - p: 0.05*) levels (Fig. 4a and d). In the heatmap graph, no significant groupings were observed between the analyzed factors (Fig. 4e).

**Fig. 5:**
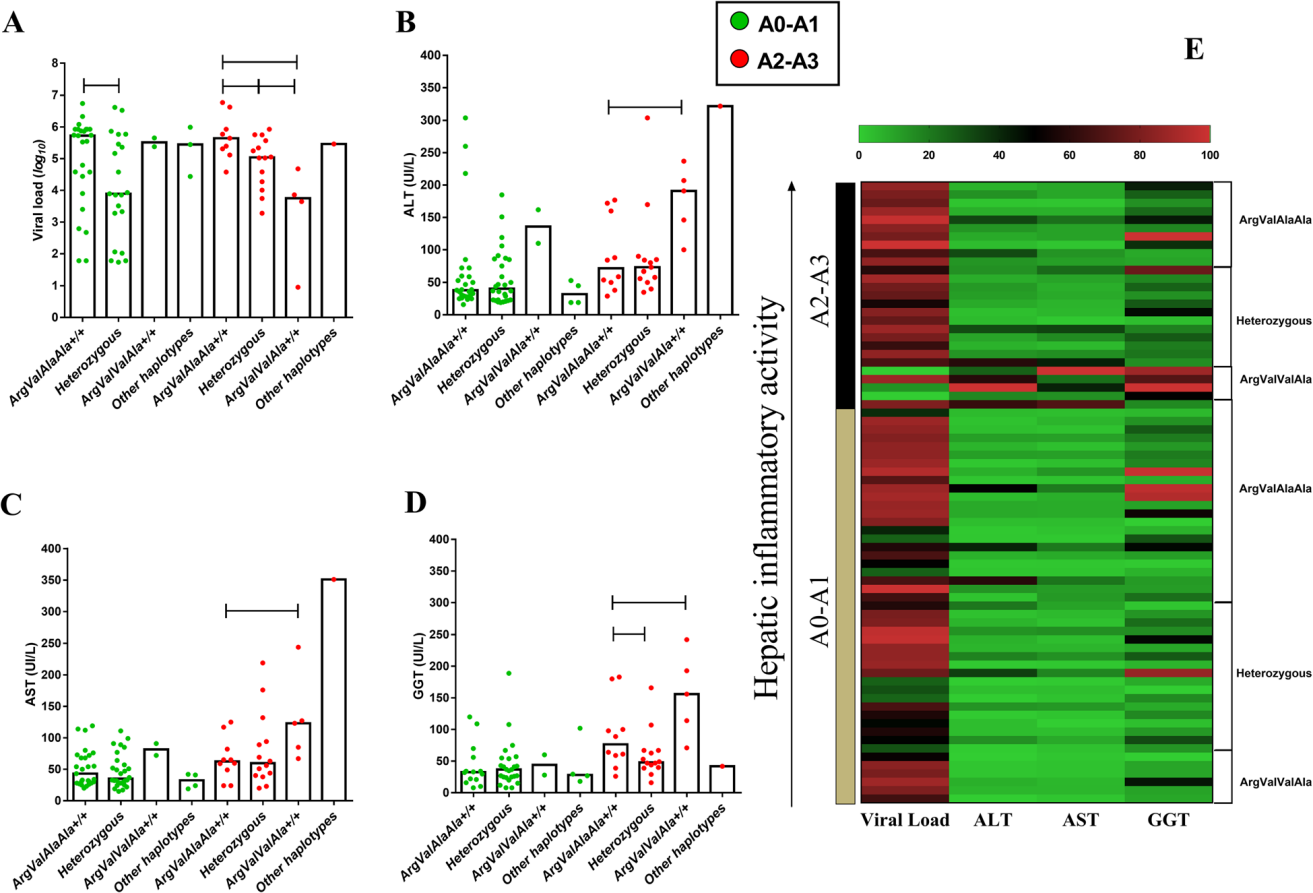
Association of liver function with NGF haplotypes according to hepatic inflammation. **a** The viral load was higher in patients with the haplotype containing the *Ala*^*+/+*^ variant at all stages of inflammation. **b**, **c**, **d** Only in A2-A3 inflammation was the level of serum markers of liver damage lower in patients with the haplotype containing the *Ala*^*+/+*^ variant. This trend was plotted on the heatmap grouping graph (**e**). Heterozygous: *ArgValAlaAla - ArgValValAla*. Other haplotypes: *ArgValAlaAla, ArgMetAlaAla, ArgValAlaAla, ArgValValAla, ArgMetValAla, ArgMetValAla, ArgValAlaAla,* and *ArgValAlaAla*

Patients with A0-A1 inflammatory activity with the *ArgValAlaAla*^*+/+*^ haplotype had the highest VL when compared to heterozygous patients (*ArgValAlaAla - ArgValValAla*) (*p: 0.0458*) (Fig. 5a). Similar results were observed in for A2-A3 inflammatory activity, for which *ArgValAlaAla*^*+/+*^ individuals also had the highest VL when compared to the heterozygous (*p: 0.0376*) and homozygous *ArgValValAla*^*+/+*^ haplotypes (*p: 0.0056*); for this classification, homozygous *ArgValValAla*^*+/+*^ individuals also had the lowest VL when compared to homozygous individuals (*p: 0.0384*) (Fig. 5a).

In contrast, for A2-A3 inflammatory activity only, individuals with the *ArgValValAla*^*+/+*^ haplotype had the highest liver enzyme activity levels when compared to individuals with the *ArgValAlaAla*^*+/+*^ haplotype (ALT - *p: 0.0193*; AST - *p: 0.0232*; GGT- *p: 0.0553*). Significant differences were also observed in the comparison of GGT between the heterozygous haplotype and the homozygous *ArgValAlaAla*^*+/+*^ haplotype (*p: 0.0019*) (Figs. 5b-d). This trend was observed in the heatmap graph, whose grouping of elevated liver enzyme levels concurred with low viral load in patients with block *ArgValValAla*^*+/+*^ and advanced inflammation (Fig. 5e).

**Fig. 6:**
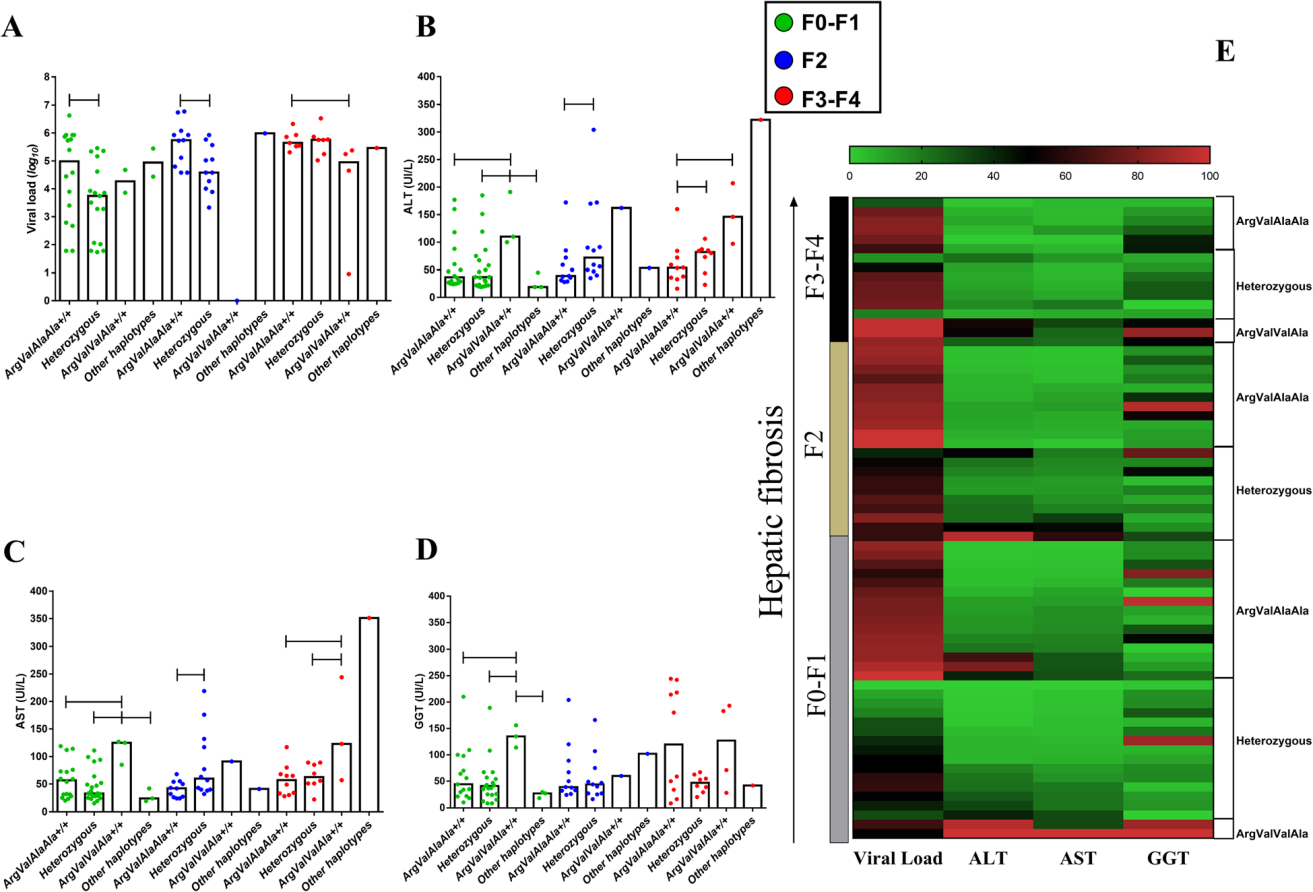
Association of liver function with NGF haplotypes according to hepatic fibrosis. **a** Viral load was higher in patients with the haplotype containing the *Ala*^*+/+*^ variant in all stages of fibrosis. **b** and **c** The level of ALT and AST was lower in patients with the haplotype containing the *Ala*^*+/+*^ variant in all fibrosis stages. **d** For GGT, this trend was observed only in the F0-F1 stage. **e** Heatmap graph showing normalized data groupings. Heterozygous: *ArgValAlaAla - ArgValValAla*. Other haplotypes: *ArgValAlaAla, ArgMetAlaAla, ArgValAlaAla, ArgValValAla, ArgMetValAla, ArgMetValAla, ArgValAlaAla,* and *ArgValAlaAla*

For the F0-F1 classification of fibrosis, individuals with the *ArgValAlaAla*^*+/+*^ haplotype had high VLs but low levels of liver enzymes compared to the heterozygous (VL - *p: 0.0473*) and homozygous *ArgValValAla*^*+/+*^ haplotypes (ALT - *p: 0.0307*; AST - *p: 0.0179*; GGT - *p: 0.0172*). Indeed, the homozygous *ArgValValAla*^*+/+*^ haplotype had the highest levels of liver enzymes among the haplotypes in this histological profile (ALT = *ArgValValAla*^*+/+*^ vs. heterozygous: *p: 0.0227*; *ArgValValAla*^*+/+*^vs. other haplotypes: *p: 0.0244*, (AST = *ArgValValAla*^*+/+*^ vs. heterozygous: *p: 0.0096*; *ArgValValAla*^*+/+*^ vs. other haplotypes: *p: 0.0054*), (GGT = *ArgValValAla*^*++*^ vs. heterozygous: *p: 0.0203*; *ArgValValAla*^*+/+*^ vs. other haplotypes: *p: 0.0009*) (Figs. 6a-d).

For the underlying stages of liver fibrosis, the results were similar to those observed for VL (F2 = *ArgValAlaAla*^*+/+*^vs. heterozygous *a*: *p: 0.0230*) (F3-F4 = *ArgValAlaAla*^*+/+*^ vs. *ArgValValAla*^*+/+*^: *p: 0.0121*) and liver enzymes, except GGT, (ALT - F2 = *ArgValAlaAla*^*+/+*^ vs. heterozygous: *p: 0.0227*), (ALT - F3 - F4 = *ArgValAlaAla*^*+/+*^ vs. heterozygous: *p: 0.0571*; *ArgValAlaAla*^*+/+*^ vs *ArgValValAla*^*+/+*^: *p: 0.0280*), (AST - F2 = *ArgValAlaAla*^*+/+*^ vs. heterozygous: *p: 0.0303*), (AST - F3-F4 = *ArgValAlaAla*^*+/+*^ vs. heterozygous: *p: 0.0212*; *ArgValAlaAla*^*+/+*^ vs. *ArgValValAla*^*+/+*^: *p: 0.0457*) (Fig. 6a-d).

The heatmap highlights the evident prevalence of low VL levels in patients heterozygotes with basal fibrosis; in the same histological profile, the highest enzyme levels grouped among the variant *ArgValValAla*^*+/+*^, while high viral load and low transaminase levels aggregated among the variant *ArgValAlaAla*^*+/+*^. In the F2 profile, we consider the tendency of grouping low VL levels with high transaminase levels among heterozygotes, the opposite is observed for patients *ArgValAlaAla*^*+/+*^ (Fig. 6e).

**Fig. 7:**
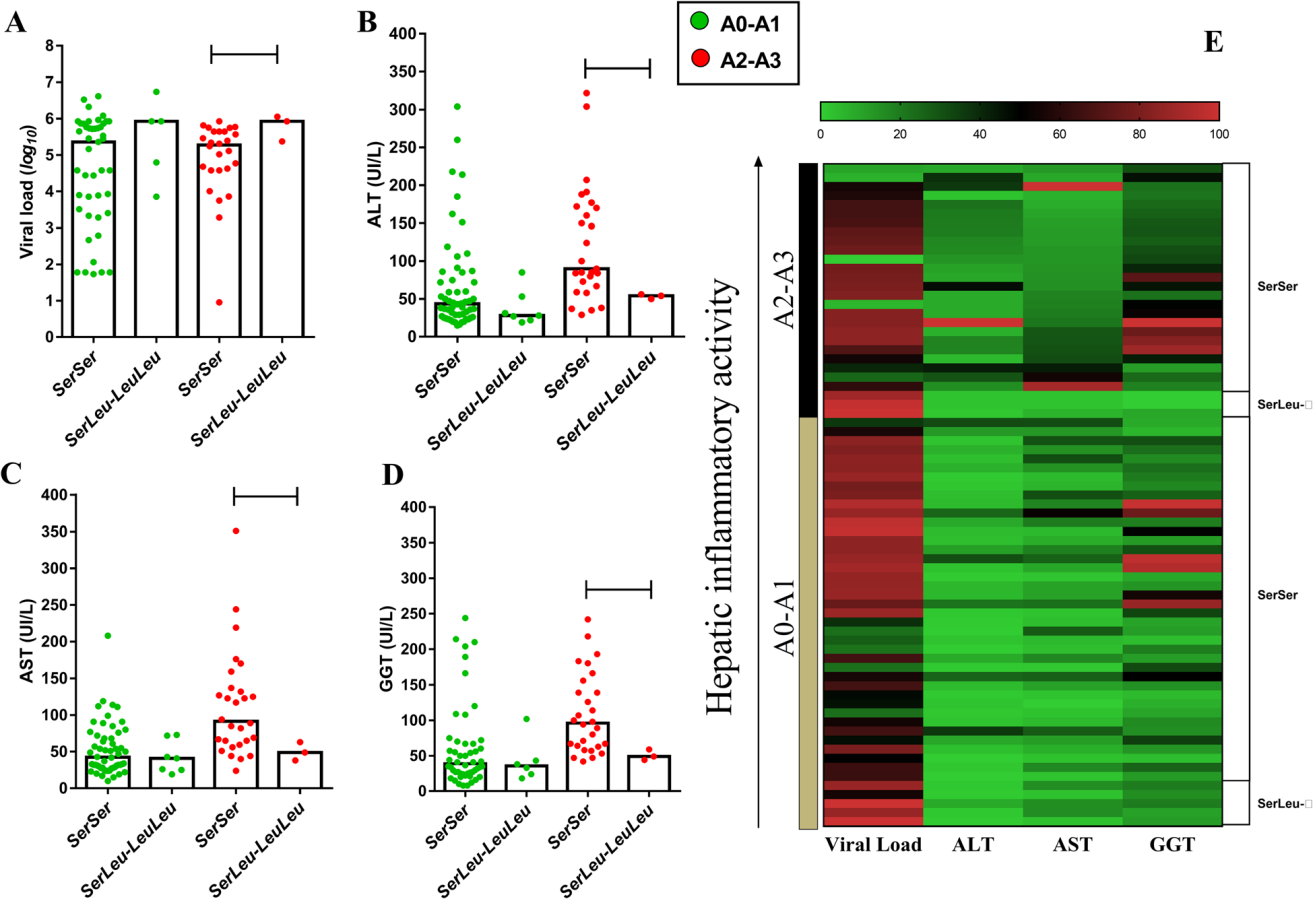
Association of liver function with *Ser205Leu* polymorphism according to hepatic inflammation. The viral load (**a**) and liver damage markers (**b**, **c** and **d**) were altered in individuals with the *SerSer* variant with A2-A3 inflammation. **e** Clustering trends were not inferred from the heatmap graph

**Fig. 8:**
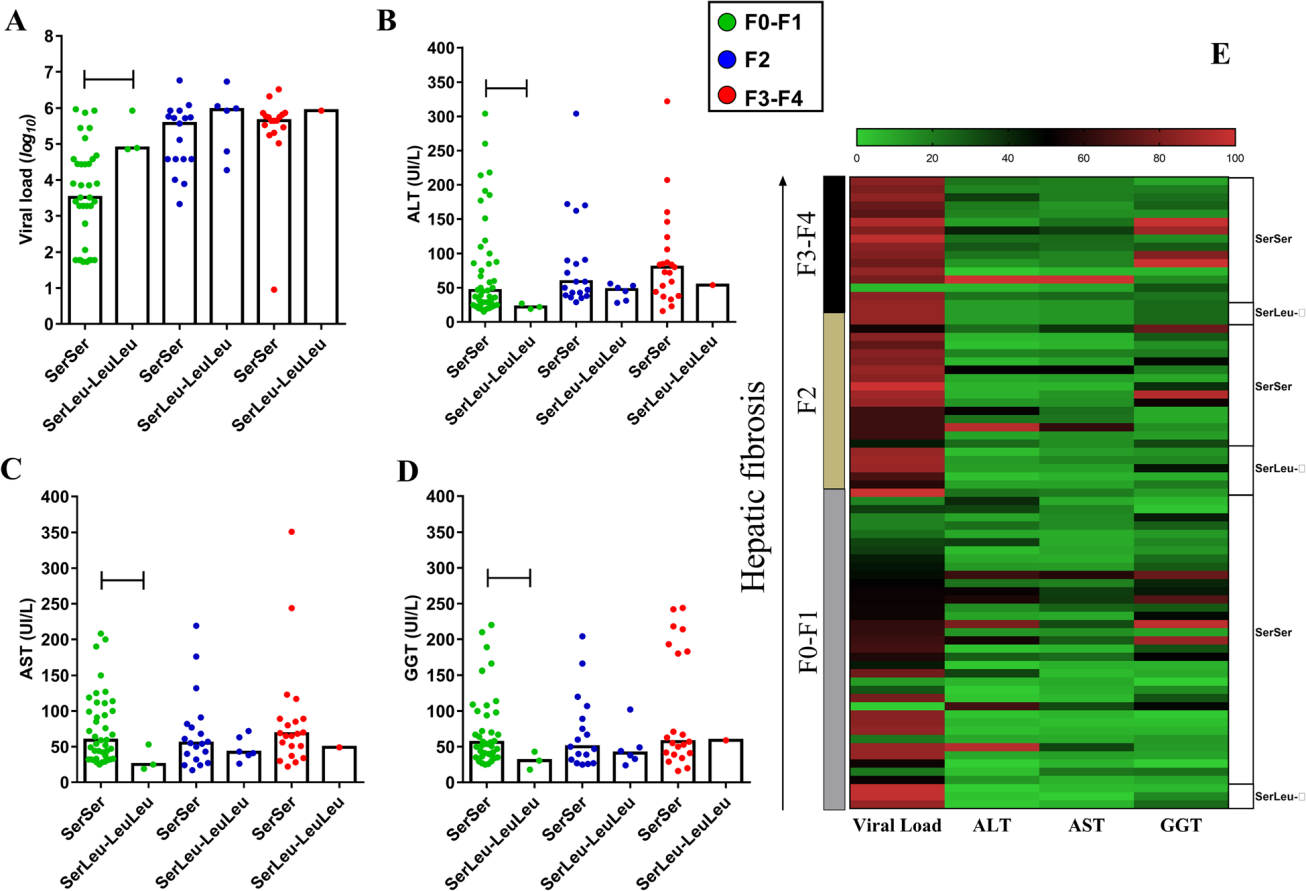
Association of liver function with *Ser205Leu* polymorphism according to hepatic fibrosis. The viral load (**a**) and liver damage markers (**b**, **c** and **d**) were altered in individuals with the *SerSer* variant with F0-F1 fibrosis. **e** Low levels of viral load prevail in the F0-F1 profile

In the evaluation of the *Ser205Leu* polymorphism in p75^NTR^, individuals with the *SerSer* variant with A2-A3 inflammatory activity had the lowest VLs but had high levels of liver enzymes when compared to individuals with the homozygous or heterozygous *Leu* variant (VL - *p: 0.0468*; ALT - *p: 0.0493*; AST - *p: 0.0440*; GGT - *p: 0.0283*) (Fig. 7). Similar data were observed for F0-F1 fibrosis (VL - *p: 0.0315*; ALT - *p: 0.0412*; AST - *p: 0.0483*; GGT - *p: 0.0470*) (Fig. 8). There were no trends of grouping according to the p75^NTR^ variants in heatmap (Figs. 7e and 8e); however, stands out the aggregate of low VL levels in fibrosis absent to mild (Fig. 8e).

**Fig. 9:**
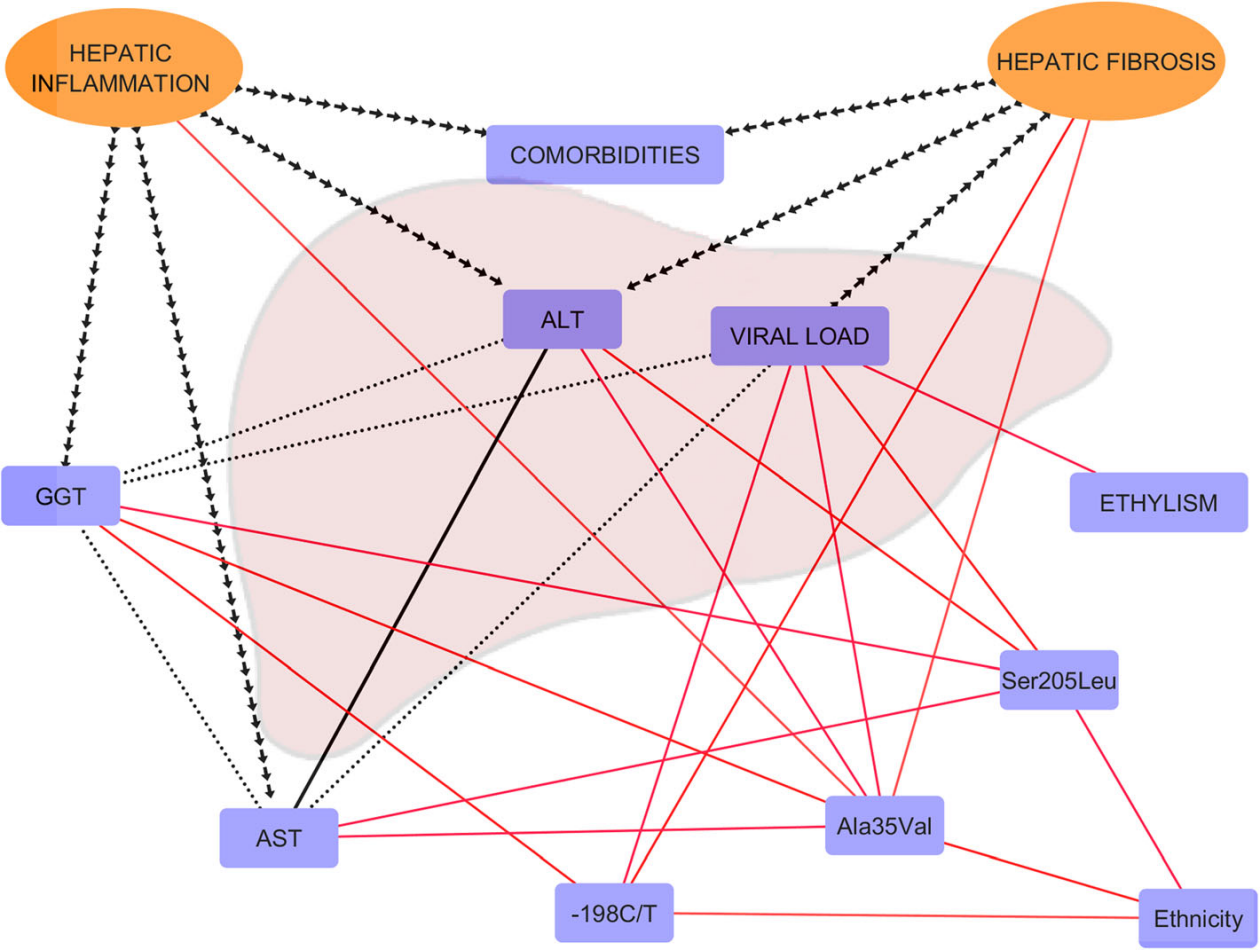
Multifactors associated with liver inflammation and fibrosis. Interaction network proposed for the relationships established. Lines shaped as separate arrows indicate the dependence of the variables, in which the origin of the arrows is the independent variables. Dotted lines indicate weak correlations between variables, based on the Pearson coefficient (*r*) (0.36 > *r*); solid lines in black indicate a strong correlation (*r* < 0.68). Solid red lines indicate significant associations between biological factors

There were positive correlations between the liver enzymes, especially ALT and AST; the correlation coefficient indicated a strong interaction between the factors (*r*: 0.8538). The plasma VL was directly proportional to the AST and GGT levels; however, the correlations between them were statistically weak (*r*: 0.2368; *r*: 0.0095). The presence of comorbidities was not associated with liver function enzyme levels. The drinking habit was a significant factor in the increase of plasma viral load (p: 0.04). The interaction network was based on the regression, association and the correlation data obtained (Fig. 9).

## Discussion

NGF and p75^NTR^ are naturally expressed in healthy liver tissue and under conditions of tissue damage (Passino et al., 2007), and their physiological roles are related to inflammation and the progression of liver fibrosis (Amoras et al., 2015). Thus, it is important to evaluate the influence of genetic variations on these factors as aspects linked to the evolution of liver damage in patients with liver disease.

Because the ***-****198C > T* SNP is an element associated with disorders (Akkad et al., 2008) and histological changes in certain pathologies (Cozza et al., 2008), it would be expected that this polymorphism could influence the hepatic microenvironment due to modulation of NGF expression induced by polymorphic variants. Notably, the gene expression dynamics of this neurotrophin are related to the regulation of liver tissue regeneration based on the stage of injury (Taub, 2004), which could be intensified by genetic variations capable of altering NGF activity. In the present study, the TT variant was associated with histological aspects and some markers of liver functionality, suggesting that NGF expression in the studied population may be dependent on this genetic factor.

Considering that the variant alters NGF gene expression (Akkad et al., 2008, Hahn et al., 2011), in the context of viral liver diseases, it is proposed that negative gene maintenance be detrimental to organ tissue integrity in the most advanced aggression profiles, which underscores the importance of NGF in controlling liver damage. In fact, in experimentally intoxicated mice, it has been shown that NGF expression by hepatocytes is directly regulated in areas of tissue regeneration during aggression (Oakley et al., 2003) (which in humans is a characteristic maintained in the advanced fibrosis stage) being pointed as a potential determinant in the resolution of the fibrotic response (Amoras et al., 2015).

In the evaluation of haplotypes of exon 3 in the *NGF* gene, the *Ala35Val* polymorphism was representative of the different haplotype profiles observed in the present study and was relevant in the analyzed processes.

In the more advanced stages of inflammation, patients with the polymorphic *Ala* variant exhibited low liver enzyme levels, indicative of liver tissue under less adverse effects. Studies emphasize the importance of neurotrophins as modulatory factors of inflammation; they can activate antiinflammatory mechanisms by regulating the presentation of antigens and controlling cytokines (Fink et al., 2014; Minnone et al., 2017). Thus, because NGF is a regulating factor of inflammatory activity, the conserved form of this neurotrophin induced by the polymorphic *Ala* variant (Cozza et al., 2008) may help in the control of advanced stages inflammation in liver tissue and, consequently, in the preservation of hepatocytes, thus leading to increased viral loads by preserving replication niches.

Regarding liver fibrosis, similar findings were observed in all stages of tissue injury, even in patients with the heterozygous *Ala* variant. Indeed, evidence suggests the proliferative role of NGF in myofibroblast cell cultures in patients with liver fibrosis (Rasi et al., 2007); activated NGF pathways contribute to the production of hepatocyte growth factors that induce the regeneration and proliferation of liver tissue in different stages of fibrosis (Passino et al., 2007). Therefore, the findings clearly show that the *Ala* variant leads to maintenance of an attenuated fibrotic response.

In contrast, the *SerSer* variant of the *Ser205Leu* polymorphism, a variant with a conserved receptor structure (Fujii et al., 2011; Gau et al., 2008), showed low viral loads and elevated liver enzyme activity levels in moderate/severe inflammation, suggesting that the wild-type p75^NTR^ pathway favors liver injury in individuals with advanced stages of inflammatory activity, as shown in different cell lines in which the p75^NTR^ pathway stimulates the production of proinflammatory cytokines that contribute to chronic tissue injury (Minnone et al., 2017; Elshaer and El-Remessy, 2017).

Adverse effects were also observed in patients with the *SerSer* variant at milder stages of fibrosis, which suggests that p75^NTR^ has a biochemically active role in fibrosis. In adults organisms, p75^NTR^ triggers the activation of Rho-kinases, which help maintain the survival of hepatic stellate cells (HSCs) and facilitate the conversion to a myofibroblast profile (Passino et al., 2007). The myofibroblasts, in turn, regulate liver fibrosis via progressive replacement of the normal parenchyma, which can result in advanced stages of injury (Novo et al., 2014). Thus, our findings suggest that the *Ser205Leu* polymorphism plays a role in this process by directly altering the viral load and liver enzymes in the initial stages of tissue fibrosis.

An interesting finding was the inverse relationship between the neurotrophin and receptor polymorphisms observed in the histopathological processes in the liver In the present study, patients with polymorphic variants associated with positive regulation, structure conservation and factor physiology showed behavior opposite for viral load and liver enzymes, indicating that NGF and p75^NTR^ do not interact with each other in the maintenance of inflammation and fibrosis at their different stages.

A range of neurotrophin receptors support interaction networks between hepatocytes, HSCs and bile duct cells (Cassiman et al., 2001), among which is the Trk receptor, which actively participates in the proliferation and function of hepatic cells (Nemoto et al., 2000). Consequently, NGF interacts with receptors such as Trk, leading to cell survival and regulation of liver tissue injury. In fact, the binding of NGF to Trk can activate intracellular signaling pathways that stimulate antiinflammatory profiles (Prencipe et al., 2014).

The paracrine loop between the neurotrophins produced by hepatocytes and the p75^NTR^ receptor expressed in HSCs, which, on the one hand, stimulates hepatocyte proliferation and, on the other hand, blocks and regulates this phenomenon (Amoras et al., 2015), was not the concept adopted in the present study. Based on the evaluation of polymorphic variants well established in the literature, we suggest that NGF has a regulatory role in maintenance of hepatocyte integrity, leading to reducing liver enzyme levels and increase of the viral load. The p75^NTR^ receptor, however, compromises the liver damage by increased the liver enzyme levels during different histopathological stages.

In historically mixed populations, it is discussed whether the complex heterogeneous structure of the population can influence the aspects of susceptibility to a particular clinical manifestation. In the Brazilian Amazonian population, integrated with the country as the largest ethnic estimates, this thought is conceivable, since it is a society with great influence of Europeans, Africans and Amerindians, with high frequency of interethnic unions historically occurring (Salzano and Sans, 2014).

In the present study, there is the level of complexity of the ethnic interactions in the population system of groups evaluate, mainly, in the varied frequency of polymorphisms associated with progression of viral liver diseases. In this aspect, the infection and the population ethnic profile are relevant factors in frequency of polymorphic variables of biological pertinence, as shown in recent studies. (Eskandari et al., 2017; Chuaypen et al., 2019).

In the analysis, it was observed that the frequency of NGF polymorphic variants related to maintenance of liver integrity was strongly associated with the African population. This could justify the average liver enzyme serum levels and a milder histopathological profile in this population. It is noteworthy that the protective alleles were emphatically associated with the progression of the liver injury, not the susceptibility to infection itself. This is consistent in assessing that in the African population the incidence and prevalence rates of chronic viral hepatitis are high, especially in men at risk of exposure and poor adherence to immunization, but with a moderate clinical course of liver disease (Crosse et al., 2004; Forde, 2017; Zuure et al., 2019).

The high frequency of polymorphic variant p75^NTR^ indirectly associated with tissue damage in the African population, a finding in apparent controversy in principle, also confirms the results of this study because we maintain that neurotrophin and receiver do not interact in maintaining the hepatic microenvironment, then it was not expected reciprocity between polymorphic frequencies.

The frequency of NGF variants, associated with the risk of progression of viral liver disease, prevailed in Europeans. Interestingly, this population is more susceptible to changes in periportal necrosis and hepatic fibrosis scores (about twice as likely, especially in patients under 40 years), as in serum transaminase levels (Crosse et al., 2004; Sajja et al., 2014). Although other studies have not identified differences in histological activity and inflammation index between Caucasians and Africans, it is notable that Caucasians had high levels of liver enzymes (Sterling et al., 2004). These results are of particular interest as European ancestry prevails in the Brazilian Amazon population (da Silva et al., 2017).

In sum, the results presented indicate that the frequency of NGF and p75^NTR^ polymorphisms are strictly related to the ethnic population aspects.

In addition to polymorphisms, other aspects of liver disease have been associated with tissue injury: Indeed, liver disease is often a reflection of biochemical abnormalities in liver function, which occurs because of repeated injury (Yang et al., 2018). Among the aminotransferases, ALT was the marker most sensitive to liver injury, which confirms the accuracy of the factor in the identification of changes in cellular integrity (Kim et al., 2008). AST, however, was related to inflammation and strongly correlated with ALT, partially in line with observations that indicate the importance of AST as a predictor of liver necroinflammation (Khattab et al., 2015). Other findings from our study also showed GGT as a marker of inflammatory activity, as proposed in chronic hepatitis, especially when there is blockage of the bile ducts (Eminler et al., 2014; Whitfield, 2001). It is considered in the risk assessment of liver fibrosis that both AST and ALT may remain normal even in cirrhosis situations (Newsome et al., 2018). GGT, in turn, is considered a serum predictor of histopathological evolution (Hu et al., 2017). However, in the present study, the high prevalence of individuals with no liver parenchymal abnormalities is suggested in the lack of association between GGT and fibrosis.

A correlation between liver fibrosis and viral load was expected (Wong, 2014); however, the positive correlation between liver enzyme levels and VL was an intriguing finding, given that in the analysis of these markers against the polymorphic variants in the different histopathological stages, an inverse relationship was observed.

The stratification of the data according to the polymorphisms allowed observation of the real effect of the different genetic variants on liver biomarkers, and in this case, viral load was a factor dependent on the integrity of the microenvironment and suppressed by local histopathological activity, as discussed in previous studies (Ito et al., 2004). On the other hand, when analyzing the unstratified data, the effect of the factors at a systematic level was observed, in which viral load contributed to the pathophysiology of liver injury through the activation of inflammation and subsequent fibrogenic activity (Nallagangula et al., 2017), which can occur long term (Li et al., 2017).

Alcoholism was not associated with the risk of histological changes, a curious finding, since it is already established that excessive alcohol consumption produces a broad spectrum of liver damage, such as steatosis, alcoholic hepatitis and fibrosis/cirrhosis, due to the ethanol metabolism produce toxic compounds that when accumulated contribute to liver fat accumulation and the substantial risk of acute liver failure (Osna et al., 2017). However, the positive correlation between alcohol consumption and viral load of aggression agents is a promising aspect that contributes to studies on the subject, since alcohol metabolism has effects on viral replication, increased oxidative stress, cytotoxicity and modulation of an attenuated immune response (Gitto et al., 2014). This offending profile can directly induce liver parenchyma modifications, favoring the increase of serum levels of the functionality enzymes, as shown in our results.

The presence of comorbidities, especially SAH, was a risk associated with progression of liver injury. There are diseases whose causal relationship with liver aggression is unclear, and the categorization as complication or comorbidity may change as further updates on the pathophysiology of liver disease evolve (Jepsen, 2014). The evaluation of SAH remains controversial in patients with advanced liver abnormalities; the prevalence of manifestation in this group is low, even in cases of renovascular disease and high circulating renin activity; patients with established arterial hypertension may become normotensive during the progression of liver disease. Future studies focus on assessing the propensity for vasodilator changes, such as hepatopulmonary syndrome, to verify the risk of this complication in patients with advanced liver disorders (Henriksen and Møller, 2004; Henriksen et al., 2006; Rajesh et al., 2009).

Diabetes is a widely studied comorbidity in advanced liver disease (Jepsen, 2014), but, similar to SAH, the interaction between these manifestations remains controversial. For the published studies, the common point observed is that diabetes, by itself, is not directly associated with mortality in patients with liver disease, this characteristic stems from a multifactorial clinical profile in which other developed comorbidities are more correlated with patient outcomes (Bianchi et al., 1994; Quintana et al., 2011). In the present study, in fact, diabetes was frequent when associated with complex manifestation profiles.

## Conclusion

In conclusion, our main results show that the presence of -*198C/T, Ala35Val* in NGF and *Ser205Leu* in p75^NTR^ polymorphisms alter hepatic functionality at different stages of inflammation and tissue fibrosis, being only the variants in NGF determinant in the establishment of histopathological profiles per se. The results emphasize the multifactorial nature of liver disease, in which the level of injury is also directly related to serum biomarkers of damage and infection; behavioral aspects and comorbidities. Although the main limitation of the present study is the sample size, this is the first to evaluate the NGF and p75NTR polymorphisms in the liver pathophysiology of patients with chronic viral liver disease in the Brazilian Amazonian population; also discussing how genetic variants are associated with ethnic diversity; a point of particular interest for heterogeneous populations. We hope that the data presented here will contribute to discussions about the role of neurotrophins and host genetic/ethnic factors in maintaining the liver microenvironment of the infected patients.

## Data Availability

The original data sets generated and analyzed during this study are made available by the corresponding author upon reasonable request. Datasets obtained from public sources are available at [dbSNP: Database for Short Genetic Variations. Available at: https://www.ncbi.nlm.nih.gov/snp/].

## Abbreviations

ALT: Alanine aminotransferase
AST: Aspartate aminotransferase
FAIM: Fas apoptosis inhibitory molecule
FSCMPA: Santa Casa de Misericórdia Foundation of the State of Pará
GGT: Gamma-glutamyltransferase
HBV: Hepatitis B virus
HSCs: Hepatic stellate cells
HUJBB: João de Barros Barreto University Hospital
JNK: C-Jun N-terminal kinase
LABVIR-ICB-UFPA: Virology Laboratory of the Biological Science Institute of the Federal University of Pará
MAF: Minor allele frequency
MAPK: MAP kinase
NF-kB: Nuclear factor kappa β
NGF: Neural growth factor
NRIF: Neurotrophin receptor interacting factor
PCHB: Patients with chronic hepatitis B
PCHC: Patients with chronic hepatitis C
PI3K-AKT: Phosphoinositide 3-kinase - protein kinase β
SNPs: Single nucleotide polymorphisms
TRAF6: Tumor necrosis factor receptor (TNFR)-associated factor 6
Trk: Tyrosine kinase
VDR: Vitamin D receptor
VL: Viral load
